# Management of Multiple Fractures in a Preadolescent With Osteogenesis Imperfecta Types III-IV

**DOI:** 10.7759/cureus.70609

**Published:** 2024-10-01

**Authors:** Saurabh Somankar, Priyansh Sahu, Anurag Luharia, Suhas Tivaskar

**Affiliations:** 1 Radiodiagnosis, Datta Meghe Institute of Higher Education and Research, Wardha, IND; 2 Radiology, Datta Meghe Institute of Higher Education and Research, Wardha, IND

**Keywords:** bone deformity, brittle bone disease, collagen, genetic disorder, osteogenesis imperfecta

## Abstract

Osteogenesis imperfecta is an inherited disorder that results in fragile bones that break easily. Gene defects are responsible for the disease. Collagen, a protein that helps strengthen bones, is produced by these genes. The disease can be classified into four types ranging from mild to lethal. Type III or type IV is the most severe forms that survive the neonatal period. In osteogenesis imperfecta strengthening the bone requires correcting the genetic mutations that cause the disorder. Physical rehabilitation, surgical procedures, and clinical management of osteogenesis imperfecta include the use of drugs such as bisphosphonates and recombinant human growth hormone. A nine-year-old male child came to the radiology department with a clinical history of bony deformities of both legs since the age of six years. The child was normal until the age of six years. Then, later, he had a right femur fracture. Surgery was done with a rod inserted in his femur, which was later removed, causing bending of his tibia and fibula. Treatment can improve the quality of life and manage symptoms, but the condition cannot be cured. As part of the treatment, bone-strengthening medications, physiotherapy, and surgery may be required.

## Introduction

A genetic disorder of mesenchymal cells, osteogenesis imperfecta, leads to generalized osteopenia, bone deformities, and also can be fracturing [1]. About 20% of all patients with osteogenesis imperfecta have type III osteogenesis imperfecta. Infants who survive the perinatal period and have fractures and deformities fall into this category. A person with osteogenesis imperfecta type III is usually diagnosed at birth because of deformities of the long bones and severe structural changes to the skeleton caused by intrauterine fractures. As far as long-term survival is concerned, type III osteogenesis imperfecta is the most severe [2]. It is common for patients with osteogenesis imperfecta type III to sustain multiple fractures during pregnancy that can be detected on an antenatal ultrasound scan. The result is that newborns have multiple long bone fractures, broken ribs, and deformed limbs [3]. Symptoms of osteogenesis imperfecta are low bone mass and bone strength, which results in fracture susceptibility, growth weakness, and bone fragility. In its various types, one out of every 15,000-20,000 births have this condition, mostly inherited in an autosomal dominant manner [4].

Osteogenesis imperfecta is a genetic disorder affecting endochondral bone ossification, leading to imperfect bone formation. To date, mutations in at least 17 different genes have been associated with osteogenesis imperfecta, including COL1A1 and COL1A2, which are the most commonly implicated. Studies are being conducted to determine whether bone quality is affected by common intracellular and extracellular pathway changes. Three cell types contribute to the formation and remodeling of bone: osteoblasts, osteoclasts, and osteocytes [5]. It is difficult to predict fracture risk in the presence of osteogenesis imperfecta despite the substantial effects on bone. Short stature and bone deformities are some of the effects of this pathology, also known as brittle bone disease [6]. An osteoblast a cell type that expresses the mutated gene product in bone is directly affected by the mutation. A change in collagen production and a change in organic and inorganic components of the bone matrix can also have an indirect effect on bone matrix composition [7]. The purpose of long-term drug therapy is to avoid complications and adverse effects treatment goals for each age group must be established due to the inability to cure this chronic disease. Centers with high levels of expertise are the best places to get molecular bone disease management [8].

## Case presentation

Patient information

We presented a case of a nine-year-old male patient who was admitted to the Medicine Department at Acharya Vinoba Bhave Rural Hospital. The patient arrived with the chief complaint of leg bending and inability to walk properly for the last three years after surgery, during which his leg became bent. The patient had no symptoms of fever, cough, cold, vomiting, redness, and pain. The patient's condition was recognized as a convex deformity of bone.

Medical/surgical history

The patient had a history of a right femur fracture at the age of six years. The child underwent successful surgery with rod insertion for bone fixation. The rod was removed after three months. Later, both the legs of the child developed convex deformity. After one year, in 2024, they came to the department with a history of osteogenesis imperfecta, and the child was completely unable to walk. There was a history of restricted movement in the right leg and no history of pain. In the left leg, there was no restriction of movement and pain. There was no history of similar complaints in the family and siblings.

Physical examination and investigations

**Figure 1:**
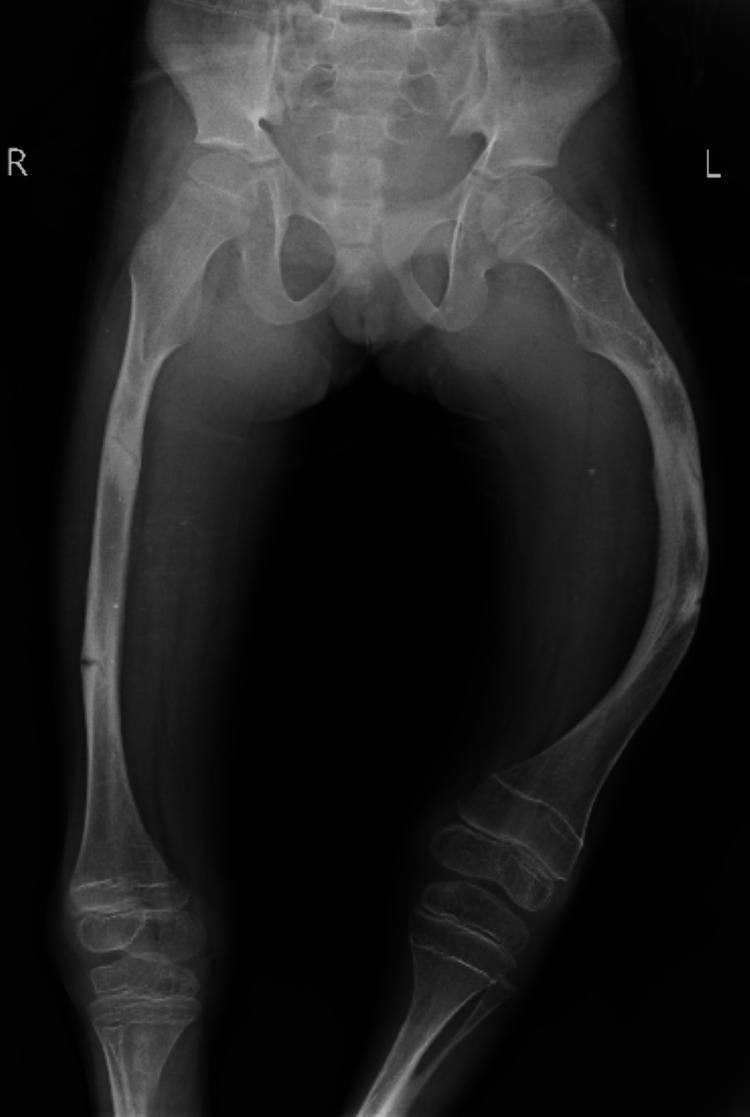
X-ray of the right femur.

**Figure 2:**
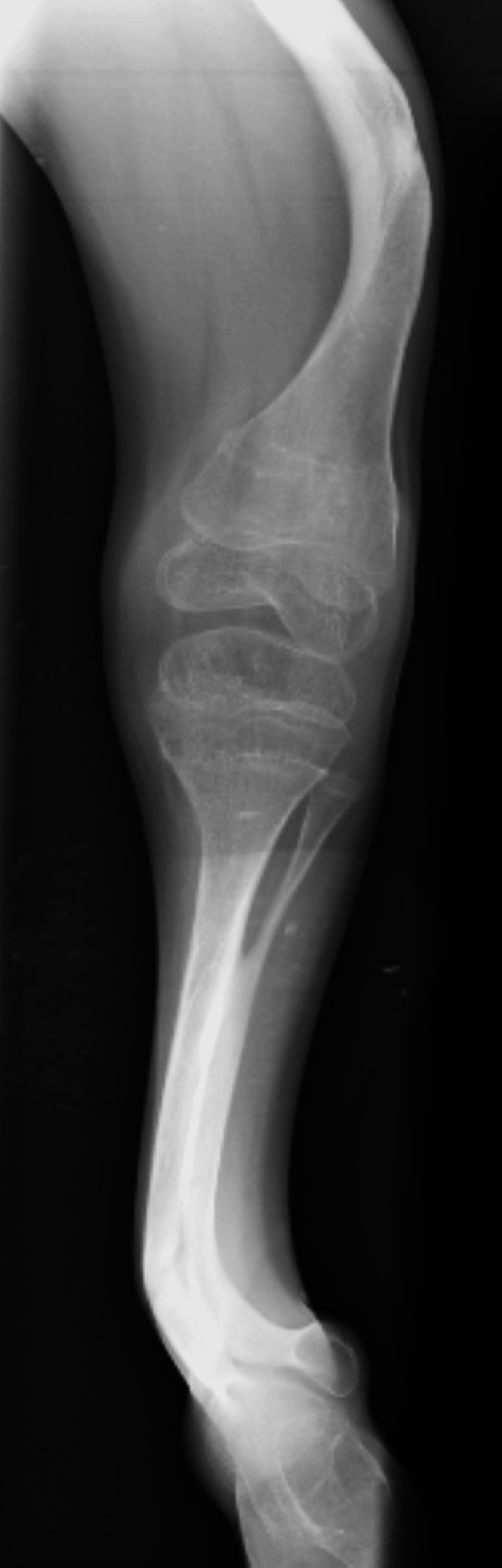
X-ray of the right tibia and fibula.

The physical examination shows the patient was febrile with an increased pulse rate of 83 bpm, an average respiratory rate of 19 bpm, a blood pressure of 100/65 mmHg, a body mass index of 14.2 kg/m^2^, a height of 120 cm and a weight of 33 kg. Pallor, clubbing, cyanosis, and pedal edema were absent. The diagnosis indicated that the patient suffered from osteogenesis imperfecta with malformation of lower limbs. The doctor advised the patient to have an x-ray of the right femur, tibia, and fibula. The lateral radiograph showed an incomplete fracture of the anterior cortex of the tibial diaphysis and bowing of the tibia. Anteroposterior radiograph of the right leg exhibited bone deformity and incurvation of the tibia and fibula, as shown in Figures 1-2.

Treatment

The patient underwent the following treatment plan for convex deformity of the leg. A treatment plan was designed to cure this condition. The patient was prescribed 5 mL of Zincovit multivitamin syrup daily, 5 mL of Calcimax syrup daily, 750 mg of Ceftriaxone injection, and 15 mg of Pantop administered intravenously each day. Osteotomy and surgical fixation with nailing for the deformity were also planned. Cyclic administration of bisphosphonate, which has become the gold standard for treatment helps in increasing bone mineral density. Following the procedure follow-up radiographs were obtained to assess the outcome.

Follow-up 

**Figure 3:**
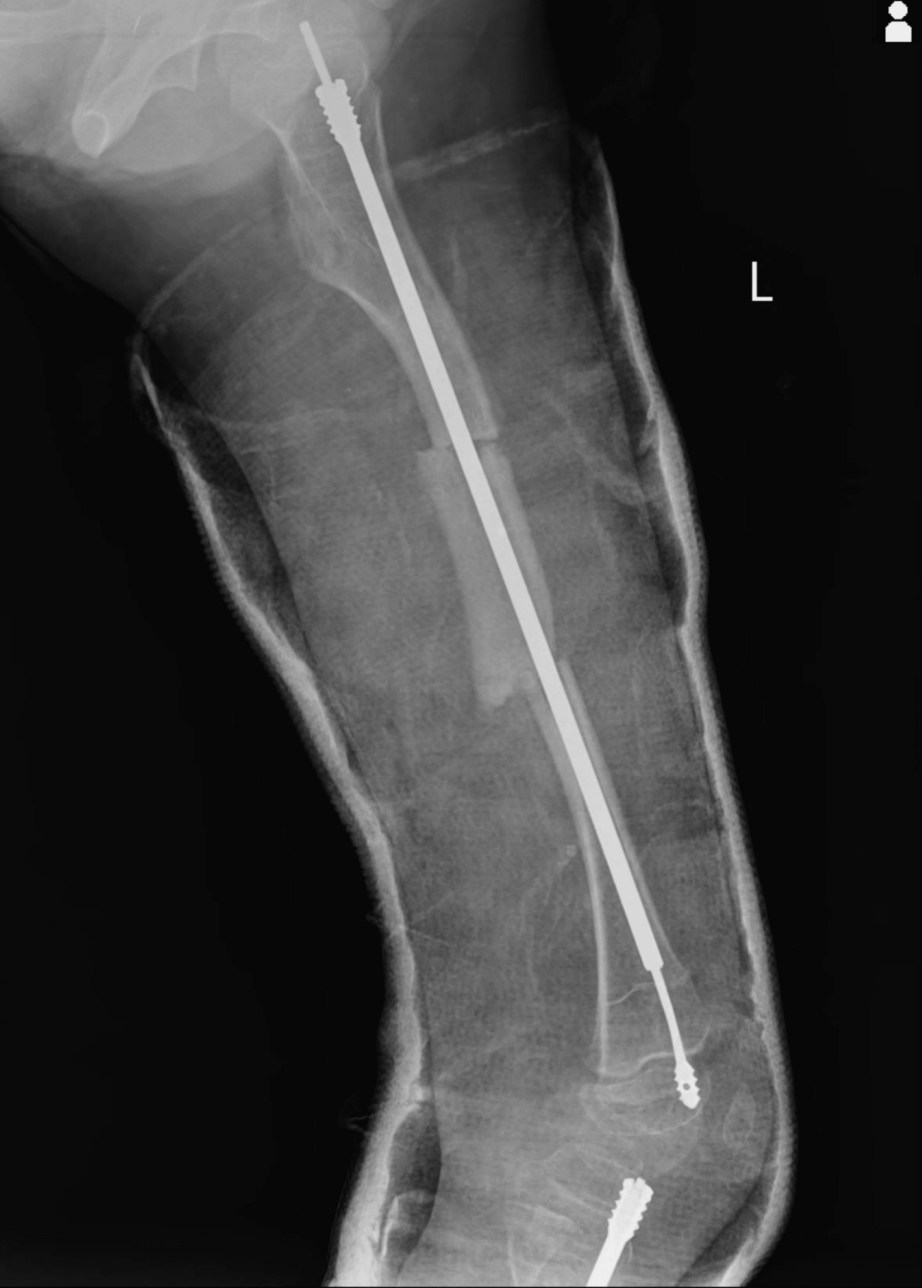
Follow-up X-ray of the patient's femur after rod implantation.

There was no complication faced by the patient throughout the procedure. The patient was advised to attend regular checkups and also prescribed regular medications including Zincovit and Calcimax syrup 5 mL twice daily. Additionally, an X-ray for follow-up was advised every three months to monitor the bone status, and rod position and also monitor pain swelling, or any signs of infection at the surgical site. We evaluate the patient's ability to move and bear weight adjusting the rehabilitation plans as needed, as shown in Figure 3. Periodic tests, including complete blood count (CBC), liver function tests (LFTs), renal function tests (RFTs), and random blood sugar (RBS) levels, were conducted to evaluate the effectiveness of the oral medications and make any necessary treatment adjustments, as shown in Table 1.

**Table 1:** Blood test parameters.

Parameter	Result	Reference range
Random blood sugar (mg/dL)	90	70-140
Complete blood count		
Hemoglobin (g/dL)	14.1	13.5-17.5
White blood cells (×10^3^/µL)	7.2	4.0-11.0
Platelets (×10^3^/µL)	312	150-450
Hematocrit (%)	43.0	38.8-50.0
Mean corpuscular volume (fL)	94.1	80-100
Mean corpuscular hemoglobin (pg)	31.0	27.5-33.2
Mean corpuscular hemoglobin concentration (g/dL)	33.5	32.0-36.0
Liver function tests		
Aspartate aminotransferase (U/L)	27	10-40
Alanine aminotransferase (U/L)	34	7-56
Alkaline phosphatase (U/L)	210	44-147
Total bilirubin (mg/dL)	0.9	0.1-1.2
Direct bilirubin (mg/dL)	0.2	0.0-0.3
Albumin (g/dL)	4.4	3.5-5.0
Total protein (g/dL)	7.6	6.0-8.3
Kidney function tests		
Blood urea nitrogen (mg/dL)	15	7-20
Creatinine (mg/dL)	1.2	0.6-1.2
Blood urea nitrogen (BUN):creatinine ratio	15.7	10:20
Glomerular filtration rate (mL/min/1.73 m²)	90	>90

## Discussion

Osteogenesis imperfecta produces osteoblasts which contribute to the improvement of bone structure and function. Osteoblasts derived from the recipient's cells expressing the genetic defect may be able to engraft mesenchymal cells in osteogenesis imperfecta patients, while even though the linear growth velocities and percent increases in total body bone mineral content differed among the three patients, the incremental gains in mineral content were comparable [1]. As part of the management of osteoporosis, orthopedic surgery plays a critical role in preventing the disuse of osteoporosis by disusing fractures that need to be reduced and immobilized for a short period with adequate bridging. Once children are ready to stand, an intramedullary rod may be placed to assist with ambulation and weight bearing. Rods may also be useful in holding the ends of a broken long bone in alignment during healing [8].

Lower limb deformities are corrected by restoring normal mechanical alignment and improving function. The next step is to decide whether to use guided growth or corrective osteotomy with acute or gradual corrections [9]. The type and stage of surgery required to treat bowing depends on the patient's age, condition, cause, and stage. Treatment aims to align the mechanical elements satisfactorily. The most common reasons for performing osteotomies are Blount disease, achondroplasia, vitamin D-resistant rickets, and osteogenesis imperfecta. A radiologist needs to assess postoperative studies to determine if a recurrence of bowing has occurred, which can be either varus or valgus. An asymmetric closure of the growth plate can also affect surgical outcomes [10].

Combined with physical therapy and timely orthopedic surgery, intravenous bisphosphonates reduce fracture rates and improve functional outcomes for children with moderate to severe osteogenesis imperfecta [11]. Bone fragility caused by reduced bone mass is the main clinical feature of osteogenesis imperfecta, leading to repetitive fractures. Patients with osteogenesis imperfecta may have stiff mineralized bones, but they break more easily under external loads. Children with large bone mass and size are more likely to suffer fractures. Fracture risk is best predicted by low bone mineral density and small bone area for height [12]. Our case highlights the challenges of managing a patient with types III and IV, which cause bone deformity, and the patient was treated with bisphosphonates and surgical implantations, which helped the patient to recover early.

## Conclusions

The management of multiple fractures in a preadolescent with osteogenesis imperfecta types III-IV presents a complex challenge that requires a multidisciplinary approach. Effective strategies include rod implantation to straighten the bones. Regular X-rays are taken to monitor bone healing and identify potential complications, such as rod migration or fractures at the surgical site. Personalized care plans ensure adequate calcium and vitamin D intake and consider bisphosphonate therapy to strengthen bone density. Genetics and clinical factors play a major role in osteogenesis imperfecta. Clinical and histological changes alone can identify and classify osteogenesis imperfecta independent of its genetic origin. We collaborate with orthopedic specialists, physiotherapists, and nutritionists to provide comprehensive care. We also offer information about the signs of complications and consider psychological support if needed. Emphasizing patient and family education is crucial for ensuring adherence to treatment protocols and addressing the psychological aspects of living with a chronic condition.
